# Case Report: Early acute kidney failure in an 11-year-old boy with Dent disease type 1

**DOI:** 10.3389/fped.2024.1428720

**Published:** 2024-11-14

**Authors:** Nicolette Murphey, Craig Authement, Paul Hillman, Samhar I. Al-Akash, Kate Richardson

**Affiliations:** ^1^ Genetic Counseling Program, University of Texas MD Anderson Cancer Center UTHealth Graduate School of Biomedical Sciences, Houston, TX, United States; ^2^ Department of Pediatrics, Division of Pediatric Nephrology & Hypertension, McGovern Medical School at the University of Texas Health Science Center at Houston (UTHealth Houston), Houston, TX, United States; ^3^ Department of Pediatrics, Division of Medical Genetics, McGovern Medical School at the University of Texas Health Science Center at Houston (UTHealth Houston) and Children’s Memorial Hermann Hospital, Houston, TX, United States

**Keywords:** Dent disease type 1 (Dent 1), *CLCN5* gene, chronic kidney disease, kidney failure, genetic, proteinuria, hypercalciuria, nephrocalcinosis

## Abstract

Dent disease type 1 (Dent 1) is a rare X-linked genetic condition which impacts kidney function and is caused by pathogenic variants in *CLCN5.* Affected males typically develop low molecular weight proteinuria, hypercalciuria, nephrocalcinosis, nephrolithiasis, and other symptoms. Kidney failure often occurs between the third to fifth decade of life. Here, we report an 11-year-old boy with Dent 1 and a severe kidney disease phenotype. The patient presented with flank pain, nocturnal enuresis, foamy urine, and increased urinary frequency. He was found to have nephrotic-range proteinuria, without hypoalbuminemia, and a significantly decreased estimated glomerular filtration rate at presentation. Further, he did not have hypercalciuria. His family history was remarkable for kidney disease among several relatives including a maternal half-brother and two sons of a maternal great aunt. Due to his symptoms and a strong family history, the patient underwent genetic testing that detected a novel pathogenic variant in *CLCN5* [c.791dup (p.Ser265Glnfs*3)]. Given the variability of symptoms among family members and the early onset of severe symptoms in this young patient compared to prior literature, we encourage genetic testing for Dent disease in similarly affected individuals.

## 1 Introduction

Dent disease is a rare X-linked condition associated with features of kidney tubular dysfunction leading to low molecular weight proteinuria (LMWP), hypercalciuria, nephrocalcinosis and/or nephrolithiasis as well as progressive kidney failure (1). First discovered in 1964, Dent disease consists of two subcategories differentiated by the causative gene, either *CLCN5* or *OCRL* (2, 3). Dent disease type 1 (Dent 1) (OMIM 300009) is caused by hemizygous pathogenic variants in *CLCN5* located at Xp11.23 (4). Dent 1 accounts for approximately 60% of Dent diseases cases with roughly 10% of these resulting from *de novo* pathogenic variants (5). Dent disease type 2 (OMIM 300555) accounts for approximately 15% of Dent disease and is attributed to pathogenic variants in *OCRL* on Xq26.1 with affected individuals often experiencing mild intellectual disability, cataracts, and/or elevated muscle enzymes in addition to the kidney findings associated with Dent disease (6). Notably, some individuals with clinical symptomology consistent with Dent disease do not have an identifiable variant in either gene (7).

The genetic etiology of Dent 1, *CLCN5,* encodes for the protein CIC-5 that is found predominantly in the proximal tubules of the kidneys and is responsible for transporting hydrogen and chloride ions, thus regulating pH levels for the proximal tubule cells to adequately function (8). When a pathogenic variant in *CLCN5* disrupts the CIC-5 protein's proper functioning, tubular proteinuria occurs as well as hypercalciuria leading to the various manifestations of Dent disease (4). Based on past studies, the most common variant types reported thus far include nonsense and missense variants (3).

Almost all patients with Dent 1 develop LMWP in addition to symptoms of hypercalciuria in 75%–90% often accompanied by elevation of 1,25-dihydroxyvitamin D and low levels of intact parathyroid hormone. The degree of proteinuria is variable with one systematic review finding that around one half of patients had nephrotic-range proteinuria without associated edema or hypoalbuminemia (9). Another study found that 52% of children had nephrotic-range proteinuria (10). Nephrocalcinosis is also observed in 75% of affected individuals and nephrolithiasis occurs in approximately 50% (11). Furthermore, nephrocalcinosis commonly appears during adolescence, though sometimes appearing in childhood (12). Notably, Burballa et al. 2023 noted that only 26.1% of their cohort comprised of both pediatric and adults had the classic triad of LMWP, nephrocalcinosis/nephrolithiasis, and hypercalciuria highlighting the phenotype variability of this condition (3). Furthermore, children with Dent 1 are more likely to present with hypercalcuria whereas adults had higher prevalence of nephrolithiasis, nephrocalcinosis, and at least stage 2 chronic kidney disease (CKD). Typically, between the third to fifth decade of life, 30%–80% of patients with Dent disease develop kidney failure, although there are reports of kidney failure presenting as late as sixties or older (6). Aside from kidney manifestations, individuals with Dent 1 may also experience symptoms of rickets, osteomalacia, and growth impairments such as growth restriction and short stature (6).

While renal biopsies cannot provide a diagnosis of Dent disease, they can provide clinical insight given that glomerulosclerosis has been reported in nearly two-thirds of biopsies. Additionally, focal global glomerulosclerosis is observed more often than focal segmental, although focal segmental is only noted in roughly 6.6% of those with Dent disease (2). A separate study of children with Dent disease who underwent biopsy for nephrotic-range proteinuria found that focal segmental glomerulosclerosis was identified in 83% of biopsies, with 60% showing interstitial fibrosis, and 70% showing tubular damage (13). Other biopsy findings can include interstitial nephritis and calcium deposits (14, 15).

As an X-linked condition, Dent 1 almost exclusively affects males with female carriers typically being asymptomatic, though up to 60% of female carriers may show very mild elevation of urinary LMWP. In rare cases, there have been reports of female carriers manifesting more extensive phenotypes including pronounced LMWP or chronic kidney disease due to skewed X inactivation (16). Currently, there are no known genotype-phenotype correlations for *CLCN5* pathogenic variants and Dent 1 has been associated with inter- and intrafamilial variable expressivity (7). Specifically, Blanchard et al. 2016 and further by Burballa et al. 2023 noted no genotype-phenotype relationship between the following features: estimated glomerular filtration rate (eGFR), calciuria, proteinuria, and plasma potassium concentration (3, 17). Our case report is of significance as we describe a young male with a novel pathogenic variant in *CLCN5* and more advanced kidney disease than observed in medical literature for individuals with Dent 1 in childhood.

## 2 Case report

The patient is an 11-year-old European male born full-term to non-consanguineous parents. His mother was 36 years old, and his father was 43 years old at the time of his birth. The proband weighed approximately 4,252 g (90th percentile) at birth and was discharged at 2 days of life with an unremarkable neonatal course. At 10 years old, he began experiencing abdominal pain and was found to have an omental infarction. He then developed intermittent right flank pain in addition to nocturnal enuresis, foamy urine, and increased urinary frequency. He had no gross hematuria, dysuria, or edema. He was normotensive, obese and had right-sided costovertebral angle tenderness.

Our patient was initially evaluated by his primary care physician in April 2022 who noted an elevated serum creatinine (sCr) and blood on a urinalysis raising concern for a glomerulonephritis. Patient's initial clinical evaluation and lab work ordered by neprhology in May 2022 and he was followed closely over the subsequent months. His urine protein-to-creatinine ratio (UPC) was elevated to 3.07 mg protein/mg creatinine (mg/mg), consistent with nephrotic range proteinuria. Prior to our evaluation, his sCr was 1.73 mg/dl–1.96 mg/dl, correlating to an eGFR of 29–36 ml/min/1.73 m^2^ and microalbuminuria of 236 mg/g, consistent with stage G3b, A2 to stage 4 CKD (18). Notably, he had persistent nephrotic-range proteinuria across multiple visits. LMWP was not performed for this patient. His retroperitoneal ultrasound showed his right kidney being 8.9 cm in length and his left kidney being 8.7 cm in length with both kidneys showing minimally increased echogenicity, but no nephrocalcinosis. Furthermore, he had normal complement levels, hemoglobin A1c, and a negative antinuclear antibody screen. He had nephrotic-range proteinuria along with an elevated urine albumin-to-creatinine ratio at 236 mg/g. His urine calcium-to-creatinine ratio was normal at 0.147 mg/mg, showing no hypercalciuria. He was started on Cholecalciferol and Calcitriol for management of his sequelae of his advanced CKD and Lisinopril for his proteinuria. Lisinopril was given for a few weeks and then stopped due to worsening of kidney function, with return to baseline after stopping therapy.

The patient does have a significant family history of various kidney diseases. His older maternal half-brother also had nocturnal enuresis and proteinuria in childhood. He underwent two renal biopsies, the first at 7 years old and the second occurring in early adolescence, both detecting scarring and glomerulonephritis. At 28 years old, he reportedly has 60% kidney function and stage 3–4 chronic kidney disease. The patient's mother had trace amounts of proteinuria detected on urinalysis and reports 70% kidney function. Furthermore, a maternal great uncle died reportedly from kidney cancer and a maternal great aunt had two sons with unspecified kidney disease, one of which died from kidney failure and the other received a kidney transplant. Given his family history, depressed kidney function and nephrotic range proteinuria at time of presentation our differential diagnosis included nephrotic syndromes such as focal segmental glomerulosclerosis and Lupus Nephritis at initial presentation.

Given the patient's symptoms, family history, and evaluation to that point he underwent a renal biopsy in June 2022 at 10 years old that revealed mild glomerulomegaly (glomeruli measuring 225 micrometers in diameter) with preservation of podocyte foot processes, 22% of glomeruli with global sclerosis, and approximately 20% interstitial fibrosis and tubular atrophy (IFTA). Further, segmental thinning of glomerular basement membrane was noted, though immunofluorescence was unremarkable and there was normal collagen staining with no basketweaving or splitting of the basement membrane on electron microscopy. The results of the biopsy raised some concern for possible Alport Syndrome given the thin basement membrane found as it is known that staining for COL4A can be negative in some patients with Alport Syndrome. This in conjunction with an inconclusive biopsy result initiated a genetics evaluation. By his presentation to genetics in September 2022, the patient was diagnosed with stage 4 chronic kidney disease with the secondary sequalae of vitamin D deficiency and secondary hyperparathyroidism. Labs from September 2022 showed elevated creatinine, blood urea nitrogen, and cystatin C, parathyroid hormone, mild microalbuminuria, and slight thrombocytosis. Additionally, the patient had mild facial dysmorphia unrelated to Dent disease and body mass index between the 95th–98th percentile.

A kidney disease panel was ordered to evaluate 401 genes, including *APOL1*. While awaiting results, the patient's kidney function rapidly deteriorated and he was admitted to the pediatric intensive care unit during an acute illness due to influenza A infection. He received three hemodialysis treatments in-patient and six more outpatient hemodialysis treatments over a 4-week period before improvement and stabilization of kidney function was observed, and dialysis was discontinued. However, he continues to have a significantly impaired kidney function overall with an eGFR of 25 ml/min/1.73 m^2^, corresponding with stage 4 CKD (albuminuria not assessed) (18). Additionally, he developed mild hypokalemia.

Resulting in October 2022, the kidney disease panel detected a novel, nonsense hemizygous pathogenic variant in *CLCN5* [NM_000084.4] at c.791dup (p.Ser265Glnfs*3) diagnosing the patient with Dent 1. The patient's mother and symptomatic half-brother underwent familial variant analysis and were both found to harbor the same *CLCN5* pathogenic variant. Given the severity of the patient's kidney disease seemingly inconsistent with the medical literature, additional genetic testing was ordered via trio whole genome sequencing. Results in September 2023 did not identify any additional variants suggesting that Dent 1 is the sole underlying genetic etiology for the patient's kidney disease. Nephrology continues monitoring the proband closely due to his kidney dysfunction.

## 3 Discussion

Dent disease can be suspected if an individual demonstrates the following three criteria: LMWP five times above the upper limit of normal, hypercalciuria (>4.0 mg calcium/kg in 24 h in adults or the 95th percentile in children, or random urine Calcium-to-Creatinine ratio >0.2 mg/mg), and at least one of the following: nephrocalcinosis, nephrolithiasis, hematuria, hypophosphatemia, chronic kidney disease, or a family history indicative of an X-linked inheritance (6). Molecular genetic testing is pertinent as it can distinguish between the two types of Dent disease allowing for appropriate surveillance and management. To our knowledge, the case reported here demonstrates one of the youngest individuals with Dent 1 in the medical literature with such an aggressive kidney phenotype, interestingly without hypercalciuria, who harbors a novel pathogenic variant (c.791dup (p.Ser265Glnfs*3) in *CLCN5*.

In addition to a novel nonsense variant in *CLCN5,* our patient presents other findings atypical of Dent disease. First is the young age of both acute kidney failure and onset of chronic kidney disease. Our patient was obese, and obesity is a known risk factor for CKD mostly by causing hyperfiltration injury, the patient's young age makes it unlikely to be a significant contributor or cause of CKD. Additionally, hypercalciuria was not present in our patient. This is likely due to significantly reduced GFR, which has been shown to be associated with lower urinary Calcium by several investigators (19, 20). Dietary factors may have also played a role, and it is possible that a 24-hour urine would have been more accurate in evaluating hypercalciuria, but that was not performed in this case.

Currently, there is no cure or specific treatment designed for Dent disease. Although thiazide diuretics can be utilized to mitigate hypercalciuria, nephrocalcinosis, nephrolithiasis, and progression of CKD (21). One small French study found that hydrochlorothiazide did not decrease urinary calcium excretion in those with a free-calcium diet in addition to not being well tolerated in those with Dent disease (17). It remains unclear if hypercalciuria contributes to kidney failure, and therefore, this therapy may not preserve kidney function (22). Angiotensin-converting enzyme (ACE) inhibitors and angiotensin receptor blockers (ARB) are used to slow down the loss of kidney function in children with proteinuria from a variety of causes including focal segmental glomerulosclerosis, though they have not been shown to be helpful for focal global glomerulosclerosis (6). A recent retrospective study found that 54% of children receiving ACE inhibitors or ARBs had a reduction in their urine microalbumin-to-creatinine ratio (10). While there is no specific therapy for Dent disease at present, investigators are evaluating gene therapy in animal models (23). Kidney transplantation is indicated for those with Dent disease who develop kidney failure with good graft and patient survival without recurrence of disease in this patient population (24). Nephrology continues to evaluate the need for a future kidney transplant with this patient given the decline in his kidney function.

What remains difficult about Dent disease in general is the wide intra- and interfamilial variability of this genetic disorder (7). Typically, males under 10 years old only manifest LMWP and/or hypercalciuria, but are most often asymptomatic at that age. This has been consistently reported in literature with He et al., 2017 detecting nephrotic-range proteinuria and hypercalciuria among all ten boys in their cohort with age of onset from 1.2 to 5.7 years old and diagnosed with Dent disease between 1.5 and 9.8 years old, with no impairment of kidney function reported (25). As a result, many individuals might go without diagnosis of Dent disease until they become symptomatic with kidney failure occurring in the third to fourth decade of life (2).

Arnous et al. 2023 investigated genotype-phenotype of Dent 1. Their data indicated that truncating variants were located throughout the gene and did not appear clustered to specific regions in the gene (26). Additionally, individuals with truncating variants in *CLCN5* experienced kidney stones earlier and a higher stone burden had a positive correlation with CKD evolution. Furthermore, it was noted that these individuals also had higher albumin excretion rates in comparison to those with non-truncating variants (23). Our patient's variant was truncating and located in the voltage ClC-5 domain and this may in part explain his more severe phenotype.

What remains unexplained is his age. In addition to our case report, there has been one report of a 9.5 year old patient developing stage 4 CKD with a p.Ser244Leu pathogenic variant in *CLCN5* (27). Chen et al. 2021 reports a 13-year-old patient with stage 4 CKD due to a pathogenic missense variant [c. 941C>T (p.S314l)] in *CLCN5* with a clinical phenotype suggestive of Bartter-like syndrome (28). This contradicts the established age range of kidney failure 30–50 years observed in the Dent disease population. Therefore, research going forward should analyze for specific genotypes that may predispose an affected individual with a more advanced and progressive kidney disease phenotype. Since our patient had advanced kidney disease at a young age with a novel *CLCN5* variant, our case serves as an example that similarly affected families should receive anticipatory guidance about expectations of Dent disease.

## Data Availability

The original contributions presented in the study are included in the article/Supplementary Material, further inquiries can be directed to the corresponding author.
